# Recovery and utilization of phosphorus from fruit and vegetable wastewater

**DOI:** 10.1038/s41598-021-04430-1

**Published:** 2022-01-12

**Authors:** Yu Qin, Huili Li, Shuanglong Ma, Kai Li, Xiaohan Zhang, Deyin Hou, Xiaoxu Zheng, Cong Wang, Ping Lyu, Shengjun Xu, Wei Zhang

**Affiliations:** 1 School of Civil Engineering, Lanzhou University of Technology, Lanzhou, 730050 China; 2 School of Civil Engineering, Kashi University, Kashgar, 844000 China; 3 College of Resources and Environmental Sciences, Henan Agricultural University, Zhengzhou, 450002 China; 4 Shenzhen Shenshui Water Resources Consulting CO., LTD, Shenzhen, 518116 China; 5 Research Center for Eco-Environmental Sciences, Chinese Academy of Sciences, Beijing, 100085 China; 6 Yangtze River Delta Research Center for Eco-Environmental Sciences, Yiwu, 322000 China

**Keywords:** Environmental sciences, Environmental social sciences

## Abstract

Excessive discharge of phosphorus into the water bodies is the key factor to cause eutrophication. The fruit and vegetable wastewater contains large amounts of phosphorus, and it may be directly discharged into water bodies, which has a great burden on the municipal sewage pipe network. Therefore, coagulation was used to remove phosphorus, recovered the phosphorus from the wastewater into the precipitate, and then the precipitate was pyrolyzed as an efficient adsorbent for phosphate removal. By comparing the adsorption effects of adsorbents (XT-300, XT-400, and XT-500) with pyrolysis temperatures of 300 °C, 400 °C, and 500 °C on phosphate in actual phosphorus-containing wastewater and simulated phosphorus-containing wastewater at different adsorbent dosage (4 g/L, 7 g/L, and 10 g/L), it was found that XT-300 had the best performance of adsorption, and the adsorption of phosphate was endothermic and obeyed the Langmuir isotherms and Elovich kinetics. The influence of pH, coexisting anions, and the structure of XT-300 revealed that the removal of phosphate was associated with electrostatic attraction, pore filling, but could not be determined whether it was related to surface precipitation. This study provides a way and method for the recovery and utilization of phosphorus in fruit and vegetable wastewater and proves that the synthetic adsorbent was an efficient phosphorus adsorbent. In the long term, we can try to use the adsorbent after phosphorus adsorption to promote plant growth in agricultural systems.

## Introduction

A high concentration of phosphorus in water bodies can pose a serious threat to the aquatic ecosystem and environmental quality. The high concentration of phosphorus-containing wastewater may be discharged into the natural environment, it's still a tough environmental problem around the world. High concentrations of phosphate can worsen the quality of aquatic ecosystems by stimulating the growth of organisms, especially algae, in the water bodies^1–3^. It also harms aquatic flora and fauna, and human well-being^4–6^. The natural recovery efficiency of phosphate is very low because phosphate is temporarily converted into precipitation and then released into water again^7^. Therefore, it is imperative to explore an efficient technology for phosphorus removal and recovery. There are many technologies had been used to reclaim phosphorus from wastewater, such as struvite crystallization^8^, membrane hybrid system^9^, algal-based biosorption^10^, calcium precipitates^11^ etc. Among them, chemical phosphorus removal would produce a large amount of sludge due to phosphorus precipitation which might lead to new pollution^12^. The biological method for phosphorus removal is sensitive to operation parameters and the efficiency is unstable^13,14^. In addition, biological treatment involves waste-activated sludge treatment or other pretreatment units, which would increase the cost of wastewater treatment. The adsorption method has low-cost and high efficiency. Therefore, it has been widely used to remove phosphate from water^15^. Bioretention system is a widely used adsorption phosphorus removal system. Its phosphorus removal efficiency is highly variable and unstable. This is mainly due to the small capacity and poor stability of phosphorus adsorbed on the filter media of the biological retention system, resulting in a high phosphorus leaching effect. Therefore, phosphorus removal can be enhanced by new filter media in the biological retention system, and the application of high-efficiency and low-cost phosphorus removal materials has also received extensive attention.

Carbon-based material is a kind of pyrolysis black carbon. Its production has many environmental benefits, such as carbon sequestration, global warming mitigation, soil quality improvement, and contaminant removal^16–18^. In recent years, because of its wide range of sources, carbon-based materials had attracted much attention. Various biomass, including agricultural waste^19,20^, forestry waste^21^, and sewage sludge, can be used as raw materials for pyrolysis to prepare adsorbents with low-cost advantage^22–24^. The moisture content of fruit and vegetable waste is usually more than 90%, and volatile solids account for more than 80% of the total solids (VS/TS). Among them, cellulose, lignin, sugars, and hemicellulose account for about 9.0%, 5.0%, and 75.0% respectively^25,26^. The conventional treatment methods of fruit and vegetable waste include incineration, landfill, aerobic composting, and anaerobic digestion, due to their high moisture content, the above methods have poor treatment effects^27–33^. In addition, in some areas, fruit and vegetable waste is crushed and dehydrated to reduce the moisture content of solid matter, and then the solid matter is composted and digested to alleviate the acid inhibition phenomenon caused by rapid hydrolysis of wastes with high moisture content during direct digestion^30,34^. The waste liquid is directly discharged into the municipal sewage pipe network and enters the sewage treatment plant together with the sewage. The treatment of fruit and vegetable wastewater is not included in the scope of design conditions in the municipal sewage system, which is easy to cause pipeline blockage, and therefore this method could not be fully popularized. Secondly, after the fruit and vegetable wastewater enters the municipal sewage collection system, it would be concentrated into the urban sewage treatment plant. Its high carbon, high nitrogen, and high phosphorus would aggravate the operation load of the sewage treatment plant. Coagulation can remove most of the phosphorus, making most of it gathered in the precipitates, and the precipitates could be prepared as adsorbents to recover phosphorus.

In this study, taking the fruit and vegetable wastewater as the matrix, most of its granular substances, especially phosphorus, are flocculated and precipitated by coagulation, and the precipitates are employed as raw materials to fabricate phosphorus adsorbent. The main purposes of this study are as follows: (1) Using the method of coagulation to coagulate and remove the high carbon, high nitrogen, and high phosphorus in fruit and vegetable wastewater, and the recovered precipitates to prepare a new phosphorus adsorbent; (2) To analyze the adsorption process by batch experiment and model analysis; (3) To explain the adsorption mechanism by various characterization. This study is expected to verify that the synthesized carbon-based material was an efficient phosphorus adsorbent, which could be used for phosphate adsorption in actual water bodies, to provide a method for the preliminary recovery and utilization of high carbon, high nitrogen, and high phosphorus in fruit and vegetable wastewater.

## Materials and methods

### Experimental instruments and reagents

The instruments used in this experiment were a tubular furnace, blast drying oven, AA3 flow analyzer, crushing press. In this experiment, polyaluminium chloride (PAC), polyferrous sulfate (PFS), polyaluminium ferric chloride (PAFC), and polyacrylamide (PAM) were purchased from Zheng zhou Senhai factory. Potassium dihydrogen phosphate, sodium hydroxide, and hydrochloric acid were purchased from Sinopharm Reagent Co., Ltd. All chemical reagents were analytically pure. The fruit and vegetable wastes were taken from the farmers' market of Changsha County, Changsha City, Hunan Province. After taking them back, the fruit and vegetable wastes were put into a crushing press for crushing and pressing, and the filtrate was taken for the experiment.

### Adsorbent preparation

To compare the coagulation effects of different coagulants on actual wastewater, conventional coagulants PAC, PFS, PAFC, and coagulant aid PAM were used to treat the wastewater, in which the coagulant solution was prepared according to 5 g:100 ml and the coagulant aid solution was prepared according to 0.2 g:100 ml.

First, compared the dosage of the three coagulants according to the dosage of 2, 4, 6, 8, and 10 g/L and the dosage of coagulant aid according to 0.04 g/L, and the precipitates under the best conditions were collected, then they were dried in a 105 °C oven.

Second, the precipitates were dried and grind with a mortar, then they were put into a tubular furnace, and were carbonized at 300–500 °C (nitrogen was used as the protective gas, and the initial temperature is 25 °C, the heating rate is 5 °C/min, which were maintained at 300 °C, 400 °C, and 500 °C for two hours respectively ). After carbonization, they were cooled to room temperature in a nitrogen atmosphere. The three materials were named XT-300, XT-400, and XT-500.

Finally, the adsorbents were cleaned with deionized water until the solution was neutral, and the adsorbents were dried in a 105 °C oven and screened with 100 mesh, then they were put into a dryer for standby.

### Batch test

In the batch experiments, three parallel were set in each group. A certain amount of potassium dihydrogen phosphate solution was dried in an oven at 105 °C for 2 h, and 4.3956 g was dissolved in 1 L deionized water to form a phosphate reserve solution of 1000 mg/L.

In order to investigate the effect of different pyrolysis temperatures on the adsorption capacity of different phosphate solutions, 0.1, 0.2 and 0.3 g XT-300, XT-400 and XT-500 adsorbents were placed into 30 mL phosphate solutions with concentrations of 0, 10, 20, 30, 70, 150 and 200 mg/L respectively. At the same time, 0.1, 0.2, 0.3 g XT-300, XT-400, XT-500 adsorbents were placed into 30 mL of supernatant after coagulation. The above solutions were shaken at 180 rpm for 24 h at 25 ± 1 °C to investigate the adsorption effect of the adsorbents on actual phosphorus-containing wastewater and simulated phosphorus-containing wastewater.

In this study, Langmuir and Freundlich’s adsorption isotherms were used to fit the data.

Langmuir model: $${q}_{e}=\frac{{q}_{m}{k}_{L}{C}_{e} }{1+{k}_{L}{C}_{e}}$$

Freundlich model: $${q}_{e}={K}_{F}{{C}_{e}}^\frac{1}{n}$$

Where q_e_ is the adsorption capacity of adsorbent per unit mass when reaching adsorption equilibrium, mg/g; q_m_ is the maximum adsorption capacity, mg/g; C_e_ is the concentration of phosphorus at equilibrium, mg/L; K_L_, K_F,_ and n are constants. In the adsorption thermodynamics experiment, 0.3 g XT-300, XT-400, and XT-500 adsorbents were placed into 30 mL of 0, 20, 40, 60, 80, 100, and 120 mg/L phosphate solution respectively, and the above solutions were shaken at 180 rpm for 24 h at 25 ± 1 °C.

In this study, four common kinetic equations were used to fit the data.

First-order kinetics: ${q}_{t}={q}_{e}(1-{e}^{{-k}_{1}t})$

Second-order kinetics: $\frac{t}{{q}_{t}}=\frac{1}{{k}_{2}{q}_{e}^{2}}+\frac{1}{{q}_{e}}t$

Elovich: $${q}_{t}=a+b \mathrm{ln} t$$

Intra-particle diffusion: ${q}_{t}={k}_{i}{t}^{0.5}+C$

 Where *q*_*t*_ is the adsorption capacity of t (mg/g); k_1_ and k_2_ are the rate constants of quasi first-order kinetic equations and quasi second-order kinetic equations respectively; t is the adsorption time (min); a and b are Elovich kinetic parameters; *k*_*i*_ is the diffusion rate constant in particles (g/mg∙min^−0.5^); C is a constant, indicating the number of boundary layers of adsorbent. The greater the C value, the influence of the boundary layer on adsorption is greater. In the adsorption kinetic experiments, 0.3 g XT-300, XT-400, and XT-500 adsorbents were placed into 30 mL of 40 mg/L phosphate solution, the above solutions were shaken at 180 rpm at 25 ± 1 °C, and the phosphate adsorption capacity was measured at 0, 60, 120, 240, 600, 960 and 1440 min respectively.

Based on the above experiments, selected the adsorbents under the best temperature conditions for pH and coexisting ions influence experiment. 0.3 g XT-300 adsorbent was placed into 30 mL of 40 mg/L phosphate solution, adjusted the pH to 2, 4, 6, 8, 10, and 12 respectively, and controlled the accuracy to ± 0.1. The above solutions were shaken at 180 rpm for 24 h at 25 ± 1 °C. In the influence experiment of coexisting ions, 0.3 g XT-300 adsorbent was placed into 30 mL of 40 mg/L phosphate solution, add 0.1 M Cl^−^, NO_3_^−^, HCO_3_^−^, Cl^−^ + NO_3_^−^, Cl^−^ + HCO_3_^−^, NO_3_^−^ + HCO_3_^−^ respectively, and the above solutions were shaken at 180 rpm for 24 h at 25 ± 1 °C.

### Analysis method

Chemical oxygen demand (COD) was measured by the Lianhua reagent method, total nitrogen (TN) was measured by alkaline potassium persulfate spectrophotometry, ammonia nitrogen (NH_3_-N) was measured by Nessler reagent spectrophotometry, total phosphorus (TP) and phosphate (PO_4_^3−^) were measured by molybdenum antimony ascorbic acid spectrophotometry, pH was measured by portable pH meter, and conductivity was measured by a conductivity meter. The specific surface area and pore structure of the adsorbents were measured by ASAP 2020HD88 physical adsorbent, the surface characteristics of the adsorbent were analyzed by ZEISS MERLIN Compact scanning electron microscope, and the diffraction pattern was analyzed by panasco X’Pert PROX diffractometer at 10°–80°. All data were plotted and analyzed by Origin and SigmaPlot, and significance analysis was conducted by SPSS.

## Results and discussion

### The phosphorus adsorption capacity of pyrolytic adsorbents at different temperatures

**Figure 1:**
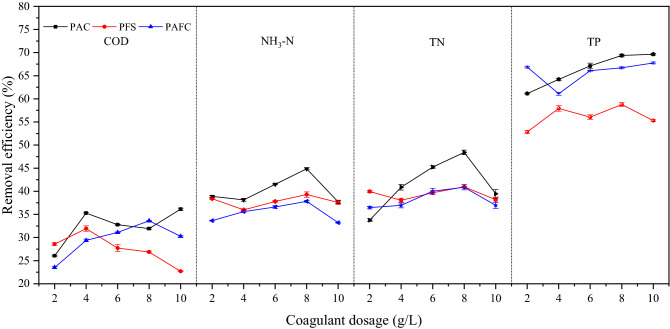
Removal efficiency of pollutants from fruit and vegetable wastewater by different coagulants.

As shown in Table 1, the water quality of fruit and vegetable wastewater was high in carbon, nitrogen, and phosphorus content and solid content. A large part of granular carbon, nitrogen, and phosphorus could be removed by coagulation. As shown in Fig. 1, the treatment effect of several common coagulants on fruit and vegetable wastewater was shown. The combination of PAC, PFS, or PAFC with coagulant aid PAM could improve the water quality. Considering the demand of preparing the phosphorus adsorbent, the precipitate produced by the combination of PFS and PAM was used to prepare the adsorbent. As can be seen from Fig. 1, when the dosage was 4 g/L, a high removal rate of phosphorus can be achieved. Therefore, the dosage of PFS and PAM finally selected in this experiment was 4 g/L and 0.04 g/L.

**Table 1:** Basic indicators of fruit and vegetable wastewater.

Index	COD (mg/L)	NH_3_-N (mg/L)	TN (mg/L)	TP (mg/L)	pH	Conductivity (ms/cm)
content	50,000–70,000	300–500	800–1200	200–400	5.0–5.5	9–10

**Figure 2:**
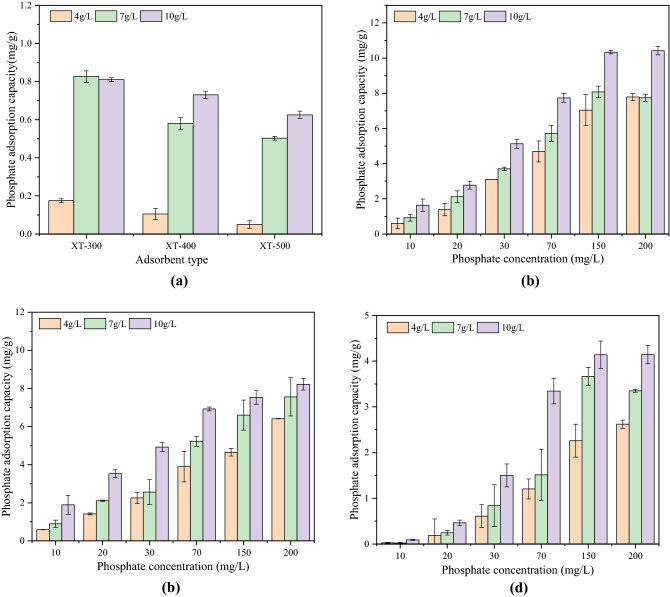
Phosphate adsorption capacity of pyrolytic adsorbents at different temperatures for actual Phosphorus-containing wastewater (**a**) and simulated phosphorus-containing wastewater (**b**) XT-300, (**c**) XT-400, (**d**) XT-500.

Figure 2 shows the adsorption effect of adsorbents at different phosphate solutions in water bodies. It can be seen that whether it was actual phosphorus-containing wastewater or simulated phosphorus-containing wastewater, the adsorption capacity of adsorbents for phosphate decreased with the increase of pyrolysis temperature. In the actual phosphorus-containing wastewater, when the dosage of adsorbent was 4 g/L, the adsorption capacity of XT-300 was 0.175 mg/g, which was greater than XT-400 (0.105 mg/g) and XT-500 (0.05 mg/g) (P < 0.05). When the dosage increased to 10 g/L, the adsorption capacity of XT-300 was 0.81 mg/g, which was still greater than XT-400 (0.73 mg/g) and XT-500 (0.625 mg/g) (P < 0.05). In addition, the phosphorus adsorption capacity of XT-300 is greater than that of XT-400 and XT-500 in simulated phosphorus-containing wastewater at either 4 g/L or 10 g/L (P < 0.05). That was, XT-300 had the best adsorption efficiency. In addition, within a certain phosphate concentration range, the adsorption capacity of adsorbents at the same phosphate concentration increased with the increase of adsorbent dosage. The reason might be that when the dosage of adsorbents was large, the adsorption sites available for adsorption increased so that the adsorption capacity of phosphate increased. At the same time, the equilibrium adsorption capacity of adsorbents increased first and then flattened with the increase of initial phosphorus concentration, which might be related to the ratio of initial phosphorus concentration to the number of available sites on the adsorbents surface^35^.

#### Adsorption isotherm

**Figure 3:**
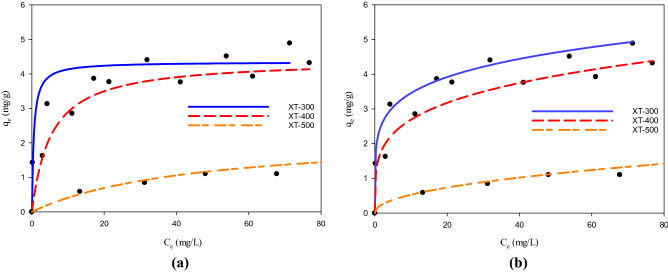
Adsorption isotherms of phosphate by adsorbents at different temperatures (**a**) Langmuir model, (**b**) Freundlich model.

The isothermal adsorption of phosphate by adsorbents at different temperatures is shown in Fig. 3, and the adsorption parameters are shown in Table 2. The amount of phosphate adsorbed by the adsorbents pyrolyzed at each temperature was positively correlated with the concentration of phosphate at equilibrium. Comparing Langmuir and Freundlich models, it could be found that the Freundlich model (R^2^ = 0.9936) could better describe the adsorption of phosphate by XT-300 than the Langmuir model (R^2^ = 0.9378), while XT-400 and XT-500 were more suitable to be described by the Langmuir model (R^2^ = 0.9896 and R^2^ = 0.8125) than the Freundlich model (R^2^ = 0.9659 and R^2^ = 0.8061). It showed that XT-300 adsorbent had an uneven surface, and its adsorption belonged to single-layer adsorption of uneven surface^36^, and the slope 1/n in Freundlich equation is an index reflecting the difficulty of adsorption. When 1/n is 0.1 ~ 0.5, it indicates easy adsorption, and when 1/n > 2, it indicates difficult adsorption^37,38^. Wu^39^ et al. reported had similar ideas. XT-300 biochar prepared in this experiment was 0.1 < 1/n < 0.25, showing that its adsorption of phosphate belonged to an easy adsorption process. XT-400 and XT-500 belonged to monolayer chemisorption with uniform surface^40^. Similar to the XT-400 and XT-500, Bulut^41^ et al. found in the study of bentonite adsorption of congo red that the adsorption also belonged to homogeneous monolayer chemisorption.

**Table 2:** Adsorption parameters of phosphorus adsorption isotherm model of adsorbents at different temperatures.

Model	Parameter	Adsorbent type			
		XT-300	XT-400	XT-500	
Langmuir	q_m_/(mg/g)	4.3474	4.4201	2.2420	
	K_L_/(L/g)	1.9295	0.1901	0.0223	
	R^2^	0.9378	0.9896	0.8125	
Freundlich	n	5.5276	4.1841	2.1037	
	K_F_/(mg/g*L^1/n^*mg^−1/n^)	2.2791	1.5529	0.1769	
	R^2^	0.9936	0.9659	0.8061	

#### Adsorption kinetics

**Figure 4:**
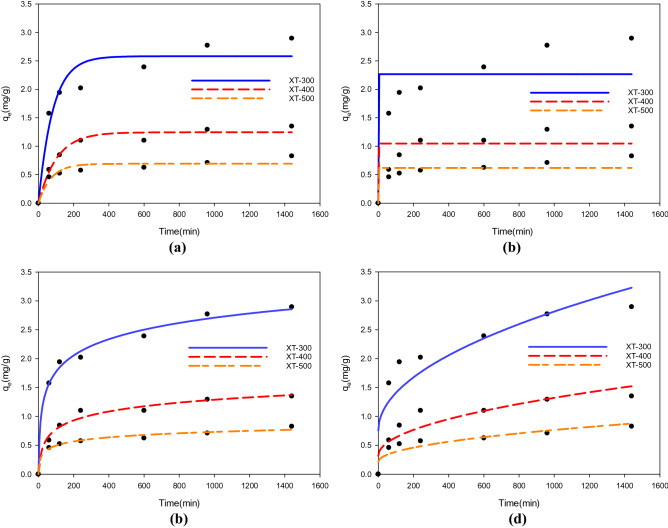
Adsorption kinetics of phosphate by adsorbents at different temperatures (**a**) First-order kinetics, (**b**) Second-order kinetics, (**c**) Elovich, (**d**) Intra-particle diffusion.

The adsorption kinetics of phosphate by adsorbents at different temperatures is shown in Fig. 4, and the adsorption parameters are shown in Table 3. It can be seen that the adsorption capacity of phosphate by adsorbents at various temperatures increased with the extension of time. The adsorption rate increased the fastest in the first 3 h and reached the adsorption equilibrium after 10 h. The solvent used in the kinetics experiment was deionized water, which was neutral and slightly acidic. The surface of the adsorbent was positively charged under such conditions. Therefore, the initial rapid adsorption might be due to the electrostatic interaction between positive charges on the surface and phosphate. In addition, in the study of Yang^42^ et al., they thought the phenomenon was possibly due to the decrease of active adsorption sites. By analyzing these four adsorption kinetic equations, it can be seen that the Elovich equation was more suitable to describe the adsorption kinetic characteristics of adsorbents for phosphate. Elovich equation is used to describe the adsorption behavior of pollutants on heterogeneous solid adsorption surfaces. It reveals the irregularity of data ignored by other kinetic equations. It describes a series of reaction mechanism processes, which are suitable for processes with large activation energy in the reaction process. However, it has no clear mechanism hypothesis for the adsorption process, but it could be found that the adsorption of phosphate by XT-300, XT-400, and XT-500 involved heterogeneous chemical adsorption, which had evenly distributed surface adsorption energy in the whole adsorption process^43^.

**Table 3:** Adsorption parameters of phosphorus adsorption kinetic model of adsorbents at different temperatures.

Model	Parameter	Adsorbent type		
		XT-300	XT-400	XT-500
First-order kinetics	K_1_/(min^−1^)	0.0122	0.0063	0.0140
	q_e_/(mg/g)	2.5818	1.4993	0.6923
	R^2^	0.9289	0.8177	0.9102
Second-order kinetics	K_2_/(min^−1^)	− 216,688	− 5,814,099	− 195,603
	q_e_/(mg/g)	2.2665	1.0466	0.6200
	R^2^	0.7709	0.6965	0.7885
Elovich	a/(g/mg/min)	0.0937	0.2456	0.0220
	b/(g/mg)	0.4058	0.2222	0.1028
	R^2^	0.9929	0.9784	0.9831
Intra-particle diffusion	K_i_/(g/mg*min^−0.5^)	0.0651	0.0317	0.0175
	C(mg/g)	0.7552	0.3190	0.2132
	R^2^	0.8122	0.8156	0.7973

#### Effect of pH and coexisting ions

According to the above finding, XT-300 had the best adsorption effect in contrast to XT-400 and XT-500. XT-300 was selected to study the effect of pH and coexisting ions on the adsorption of phosphate.

**Figure 5:**
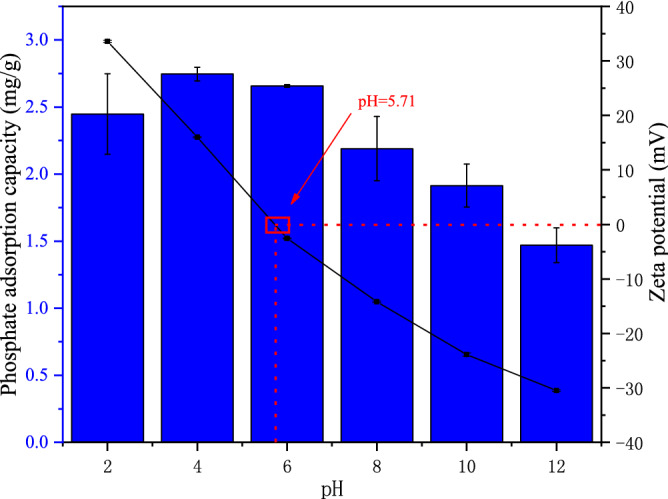
Effect of pH on XT-300 adsorption and zeta potential.

The effect of different pH on phosphate adsorption by XT-300 is shown in Fig. 5. The pH value of the solutions affects the forms of phosphorus in water bodies, and also affects the structure and chemical properties of the material surface and the charging of surface oxides. To study the effect of pH on the adsorption capacity, the pH_pzc_ of XT-300 was measured to be 5.71. It can be seen from the figure that when the pH increased from 2 to 6, the phosphate adsorption capacity of XT-300 gradually increased, and when the pH increased, the phosphate adsorption capacity of XT-300 decreased sharply. In the pH ranges of 2–6, the main phosphate anions formed are monovalent H_2_PO_4_^−^ and divalent HPO_4_^2−^ ^44^. Therefore, the positively charged surface of XT-300 was more likely to adsorb negatively charged phosphate anions H_2_PO_4_^−^ and HPO_4_^2−^, then when the pH is 2–6, the higher adsorption capacity of phosphate was attributed to the stronger electrostatic attraction. However, when pH > 6, the main forms of phosphate are HPO_4_^2−^ and PO_4_^3−^. At this time, the surface of XT-300 had negative charges, which strongly repulse the main phosphate species HPO_4_^2−^ and PO_4_^3−^. The electrostatic attraction would turn into electrostatic repulsion, ligand exchange would be inhibited, which might also lead to a decrease in phosphorus adsorption^45,46^. That was, with the increased pH value, the surface of XT-300 was negatively charged, which intensified the electrostatic repulsion between phosphate and XT-300, resulting in a poor adsorption effect on phosphate. In addition, too high pH would cause OH^−^ and PO_4_^3−^ to compete for the active sites on the surface of the adsorption material, and the surface precipitation would be weakened, resulting in the decrease of phosphorus adsorption^47–49^.

**Figure 6:**
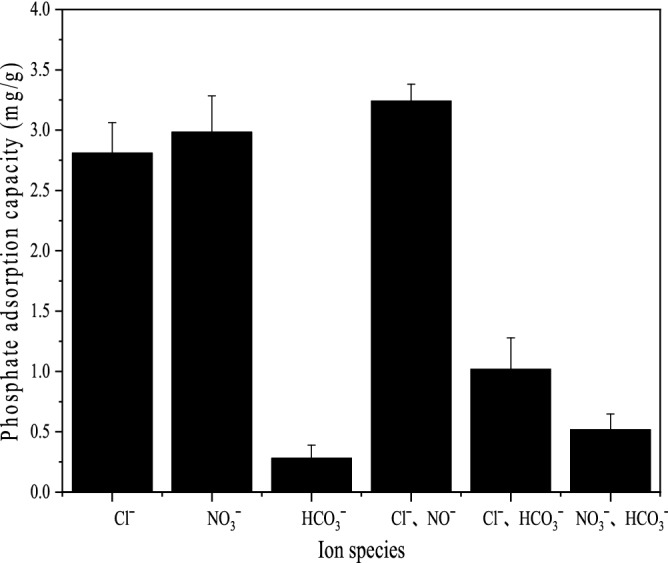
Effect of anion on XT-300 adsorption.

In addition to pollutants, there are also a large number of ions in actual water bodies. In this study, phosphate was an anion. Therefore, common anions Cl^−^, NO_3_^−^ and HCO_3_^−^ were selected to explore the effect of anions in solution on phosphate adsorption by XT-300. The results are shown in Fig. 6. It can be seen that Cl^−^ and NO_3_^−^ had no significant effect on the adsorption of phosphate by XT-300, but the adsorption capacity decreased greatly in the presence of HCO_3_^−^, especially when HCO_3_^−^ existed alone, which had a stronger inhibition on the adsorption of phosphate by XT-300, indicating that HCO_3_^−^ might compete with phosphate for the adsorption position on XT-300. This phenomenon can be explained by the that both the structures of HCO_3_^−^ and H_2_PO_4_^−^ are tetrahedral, and therefore it would produce competitive adsorption on some specific sites, resulting in poor adsorption effect^49^. That was similar to the result reported by Yang^50^. However, the effects of Cl^−^ and NO_3_^−^ on phosphate adsorption were not obvious. The reason might be that the adsorption of Cl^−^ and NO_3_^−^ belonged to nonspecific adsorption which was ascribed to the outer complex, so it would not interfere with phosphate adsorption^51^. A similar report could be seen in the study by Loganathan^7^ et al.

#### Adsorption mechanism

**Figure 7:**
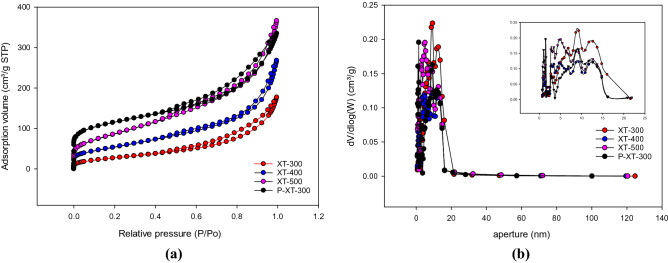
BET analysis of adsorbents at different temperatures (**a**) N_2_ adsorption/desorption isotherm, (**b**) Pore size distribution.

Before and after phosphate adsorption, the surface of biochar will have corresponding physical and chemical changes. BET characterization results of biochar at different temperatures were shown in Fig. 7. N_2_-adsorption/desorption isotherms were all the IV type. The existence of type IV isotherm indicated that the material had mesoporous distribution. The characteristics of the hysteresis loop corresponded to specific pore structure information, and the existence of the H3 hysteresis loop indicated that the mesoporous pore size was uneven. It can be seen from Table 4 that with the increase of pyrolysis temperature, the specific surface area of adsorbent gradually increased, the total pore volume gradually increased and the average pore size gradually decreased, indicating that new mesoporous channels might be formed during pyrolysis, increasing total pore volume. But at the same time, due to the increase of specific surface area, the average pore size finally decreased. In the structure of the biochar, only a small number of micropores could directly lead to the external surface of particles, and the vast majority of pore structure in the following mode distribution within the particles: Macropores opening directly on the particles of the surface of the external, mesopores would like the branches of the "growth" from the Macropores’s pore canal, and microporous then is like the branches from the mesopores in the channel of "growth". From the above adsorption phenomena, it could be seen that the adsorption effects of XT-300, XT-400, and XT-500 were gradually getting worse, and their average pore diameters were 8.46 nm, 7.45 nm, and 6.38 nm respectively, and the average pore size of XT-300 decreased after adsorption, indicating that the adsorption of phosphorus was preferentially filled in macropores and large mesopores, as a result, the pore size of macropore decreased, and the number of micropores and mesopores increased. Because there were about 80% of the total specific surface area value of biochar was contributed by micropores^52^, the average pore size of XT-300 decreased and the specific surface area increased after adsorption, and pore-filling was one of the adsorption mechanisms^51^. In the study of Sang^53^ et al., the modified biochar prepared was preferentially filled in micropores, so the average pore size decreased after adsorption.

**Table 4:** BET analysis of phosphorus adsorption by adsorbents at different temperatures.

Sample	Total specific surface area/(m^2^/g)	Mesoporous area/(m^2^/g)	Total volume/(m^3^/g)	Average aperture/(nm)
XT-300	101.51	98.36	0.26	8.46
XT-400	194.85	192.13	0.39	7.45
XT-500	312.13	307.17	0.57	6.38
P-XT-300	392.93	384.36	0.50	6.22

**Figure 8:**
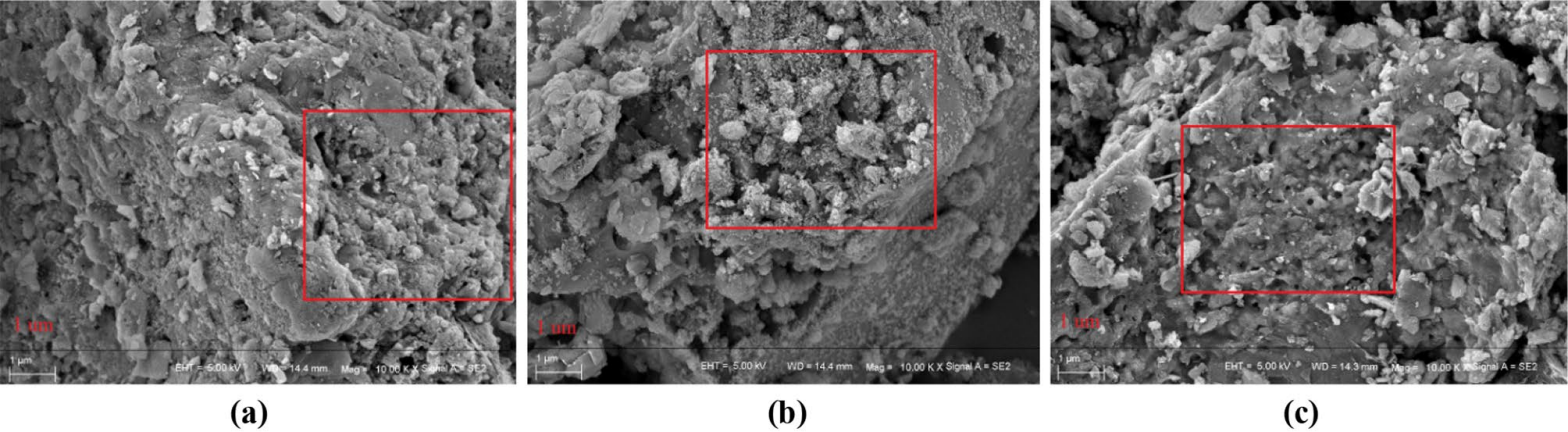
Scanning electron microscope of adsorbents at different temperatures (**a**) XT-300, (**b**) XT-400, (**c**) XT-500.

**Figure 9:**
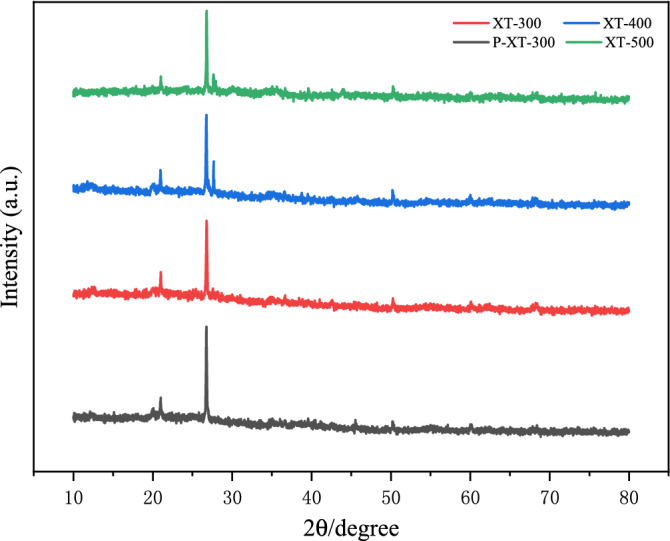
X-ray diffraction patterns of adsorbents at different temperatures.

It can be seen from Fig. 8 that XT-300 had the largest pore size compared with XT-400 and XT-500, which was consistent with the conclusion that XT-300 had the best adsorption performance for phosphorus, that was, phosphorus filled in the macropore and mesopore first. As can be seen from Fig. 9, there was a strong diffraction peak near 25°, which could be determined as SiO_2_ crystal by comparison^54^. And there was no obvious peak between 30° and 70°, indicating that iron existed in the forms of amorphous phase in the adsorbent, which was more conducive to the adsorption of phosphate than crystalline iron oxide^55^. No significant enhancement of characteristic peaks was observed after adsorption. In addition, the complex structure of fruit and vegetable wastewater might also lead to the superposition or cancellation of its characteristic peaks^56^. Therefore, it was impossible to determine whether precipitation occurred during the adsorption process. The adsorption did not change the spectrum, manifesting that the adsorption of phosphorus would not change the crystal structure of the adsorbents, and would not exist on the surface in the form of precipitation crystallization.

#### Thoughts on the reuse of adsorbent

The adsorbents in this paper were prepared by thermal hydrolysis of precipitates produced by coagulation of fruit and vegetable wastewater, and their material composition was complex. However, in the process of biochar preparation at high temperatures, various substances were immobilized in the crystal structure formed by its pyrolysis to prevent precipitation during use. In addition, Kholoma^57^ et al. found that some phosphorus would be released from biochar when it was adsorbed and reused. Therefore, the risk of phosphorus leaching should be fully considered, and biochar could be adsorbed and reused in agricultural systems to recover phosphorus. Wang^58^ et al. found that after phosphorus adsorption by biochar, seed germination rate increased from 66 to 92%, and phosphorus adsorption by biochar was dissolved and released by Pseudomonas aeruginosa to promote plant utilization. In this study, phosphorus in fruit and vegetable wastewater was recovered to precipitates by coagulation, and the precipitates were thermalized to prepare biochar, which could also be used as an adsorbent to adsorb phosphorus. In future studies, further dissolution of the adsorbed phosphorus could be considered for use in agricultural systems to promote plant growth.

## Conclusions

In this study, part of carbon, nitrogen, and phosphorus in fruit and vegetable wastewater were successfully precipitated by the combined coagulation of PFS and PAM, in which the removal of phosphorus reached more than 50%, and the recovery of phosphorus was effectively realized. It was found that XT-300, XT-400, and XT-500 had the performance of adsorption on phosphate removal, and XT-300 had the best performance of adsorption. The adsorption isotherm and adsorption kinetic experiments of the three adsorbents were carried out. It was found that XT-300 conformed to the Freundlich adsorption isotherm, while XT-400 and XT-500 were more conformed to the Langmuir adsorption isotherm, indicating that XT-300 adsorbent had an uneven surface, and its adsorption of phosphate belonged to monolayer adsorption with an uneven surface, while XT-400 and XT-500 belonged to monolayer chemical adsorption with a uniform surface. The kinetic analysis showed that the Elovich equation was more suitable to describe the adsorption kinetic characteristics of phosphate by XT-300, XT-400, and XT-500. The adsorption of phosphate involved heterogeneous chemical adsorption and had uniformly distributed surface adsorption energy in the whole adsorption process. The above analysis results showed that XT-300 had the best effect on phosphate adsorption. Therefore, the effect of pH and coexisting anions on its adsorption was investigated. It was found that there was no significant difference in adsorption when pH < 6, and Cl^−^ and NO_3_^−^ had no significant effect on its adsorption, but the adsorption capacity decreased significantly in the presence of HCO_3_^−^. Further analysis showed that the phosphate adsorption process of XT-300 involved void filling, electrostatic attraction, and could not be determined whether it was related to surface precipitation, so it needs further research to verify. This study can provide a novel method for the recovery and utilization of phosphorus from fruit and vegetable wastewater. In future research, we can try to use the adsorbent after phosphorus adsorption to promote plant growth in agricultural systems.
